# Association Between Sex and Prehospital Delay in Patients With Acute Myocardial Infarction: A Cross-Sectional Analysis of the National Hospital Ambulatory Medical Care Survey, 2006-2020

**DOI:** 10.7759/cureus.111424

**Published:** 2026-06-24

**Authors:** Moses C Odoeke, Bisola Babatunde, Akinyele Oladimeji, Steve Okwu, Vatalie Caesar, Anulika Anyata

**Affiliations:** 1 Internal Medicine, University of Toledo, Toledo, USA; 2 General Medicine, Lagos University Teaching Hospital, Lagos, NGA; 3 Family Medicine, Alberta Health Services, Edmonton, CAN; 4 Cardiothoracic Surgery, Beacon Hospital, Dublin, IRL; 5 Internal Medicine, University of the West Indies, Port of Spain, TTO; 6 Community Medicine, Alex Ekwueme Federal University, Ndufu-Alike, Abakaliki, NGA

**Keywords:** acute myocardial infarction, emergency department, healthcare access, national hospital ambulatory medical care survey, nhamcs, prehospital delay, sex differences

## Abstract

Background: Prehospital delay in acute myocardial infarction (AMI) remains an important factor affecting timely care. Differences in delay by sex have been reported, but findings are not consistent across studies. Understanding these patterns using nationally representative data is important for clinical and public health planning.

Objective: The objective of this study was to evaluate the association between sex and delayed presentation, approximated using triage acuity at emergency department arrival, among patients with AMI using nationally representative emergency department data.

Methods: A cross-sectional study was conducted using the National Hospital Ambulatory Medical Care Survey (NHAMCS) from 2006 to 2020. The study included 1,229 adult visits, representing 6,344,820 weighted emergency department visits. Prehospital delay was defined using triage acuity. Survey-weighted logistic regression was used to assess the association between sex and delay, adjusting for age category, race and ethnicity, and insurance type.

Results: Female patients had lower odds of delayed presentation compared to males, with an adjusted odds ratio of 0.69, a 95% CI of 0.51 to 0.93, and a p = 0.016. Insurance type was associated with delay, with lower odds observed among privately insured patients (adjusted odds ratio 0.52, 95% CI 0.32 to 0.85, p = 0.009) and other insurance (adjusted odds ratio 0.42, 95% CI 0.26 to 0.69, p < 0.001). Age and race were not significantly associated with delay.

Conclusion: Prehospital delay varies by sex and insurance status. These findings support continued efforts to improve timely presentation and address barriers to care.

## Introduction

Cardiovascular disease is the leading cause of morbidity and mortality worldwide, and acute myocardial infarction (AMI) remains a major contributor to adverse health outcomes, healthcare utilization, and premature death [1, 2]. The management of AMI is critical due to the nature of its etiology being time-dependent: without treatment within hours of onset, myocardial ischemia rapidly progresses to irreversible damage; therefore, the timing of treatment affects prognosis, so treatment and awareness must occur promptly [3, 4]. If patients arrive on time at the hospital, they will receive a rapid reperfusion workup with percutaneous coronary interventions or thrombolysis, thereby reducing infarct size, maintaining normal ventricular function, and improving overall outcomes [5]. Therefore, delays associated with AMI treatment must be minimized in order to improve outcomes for patients with AMI [6].

The time between the start of AMI symptoms and arrival at the hospital is considered prehospital delay, which is modified, and it is the longest part of the overall time that an individual experiences ischemia due to an AMI [7, 8]. It is known that longer prehospital delay time is directly related to greater risk of adverse outcomes, including increased death rate, greater myocardial damage due to the infarct, greater risk for mechanical complications of the heart after the infarct, and decreased effectiveness of timely reperfusion treatment [9, 10]. Since delay in seeking medical care from the onset of AMI symptoms has been shown to be strongly correlated to both treatment effectiveness and survival, improvement in the prehospital delay period will be a major goal of many initiatives in cardiovascular care, quality improvement, and public health as they relate to improving AMI outcomes [11, 12].

Persistent clinically significant sex-based differences have been reported in presentation patterns and treatment-seeking behaviors among patients with AMI. Female patients comprise a substantial proportion of the affected population, who tend to present later to the hospital than male patients after the onset of symptoms [13,14]. Additionally, females tend to report fewer classic or easily recognized symptoms of AMI (chest pain) and instead report other symptoms, such as shortness of breath, fatigue, nausea or vomiting, back pain, or general malaise and weakness [15,16]. These differences are likely caused by a combination of biological differences, sociocultural influences, misinterpretations of symptoms as an AMI, and a biased healthcare system that may contribute to delayed diagnosis and a decreased amount of time from diagnosis to receiving evidence-based treatment [17, 18].

Sex differences in prehospital delay of those with AMI have been previously investigated, yet their findings are not consistent across studies or populations [19]. Most of the previously published analyses have reported small sample sizes, were conducted at one centre or region, and were not fully adjusted for all symptoms that accompany an AMI, leading to uncertainty [20]. The National Hospital Ambulatory Medical Care Survey (NHAMCS) was used in this study as a nationally representative dataset of emergency department visits in the United States, allowing for the examination of clinical and demographic patterns across diverse healthcare settings [19]. Its broad coverage and standardized data collection provide a suitable framework for assessing differences in prehospital delay among patients with AMI. The dataset enables evaluation of sex-based patterns while accounting for key patient characteristics, offering a national perspective on variation in presentation to care [20].

Currently, there is limited understanding of how sex influences prehospital delay after accounting for demographic factors. Improving the timeliness of recognition and reducing inequities in AMI requires a clearer understanding of this relationship. This study aims to evaluate the association between sex and a study-defined measure of prehospital delay, approximated using triage acuity at presentation, among patients with AMI using nationally representative NHAMCS data, while adjusting for key demographic characteristics.

## Materials and methods

Study design and data source

This study employed a cross-sectional design using data from the NHAMCS emergency department files from 2006 through 2020 [21]. NHAMCS is a nationally representative survey of visits to non-federal, general, and short-stay hospitals in the United States, conducted by the National Center for Health Statistics. The survey uses a multistage probability sampling design that incorporates stratification, clustering, and weighting to generate nationally representative estimates of emergency department utilization. Data from all available years were appended into a single dataset, and variable names were standardized across years to ensure consistency in coding and interpretation.

Study population

**Figure 1:**
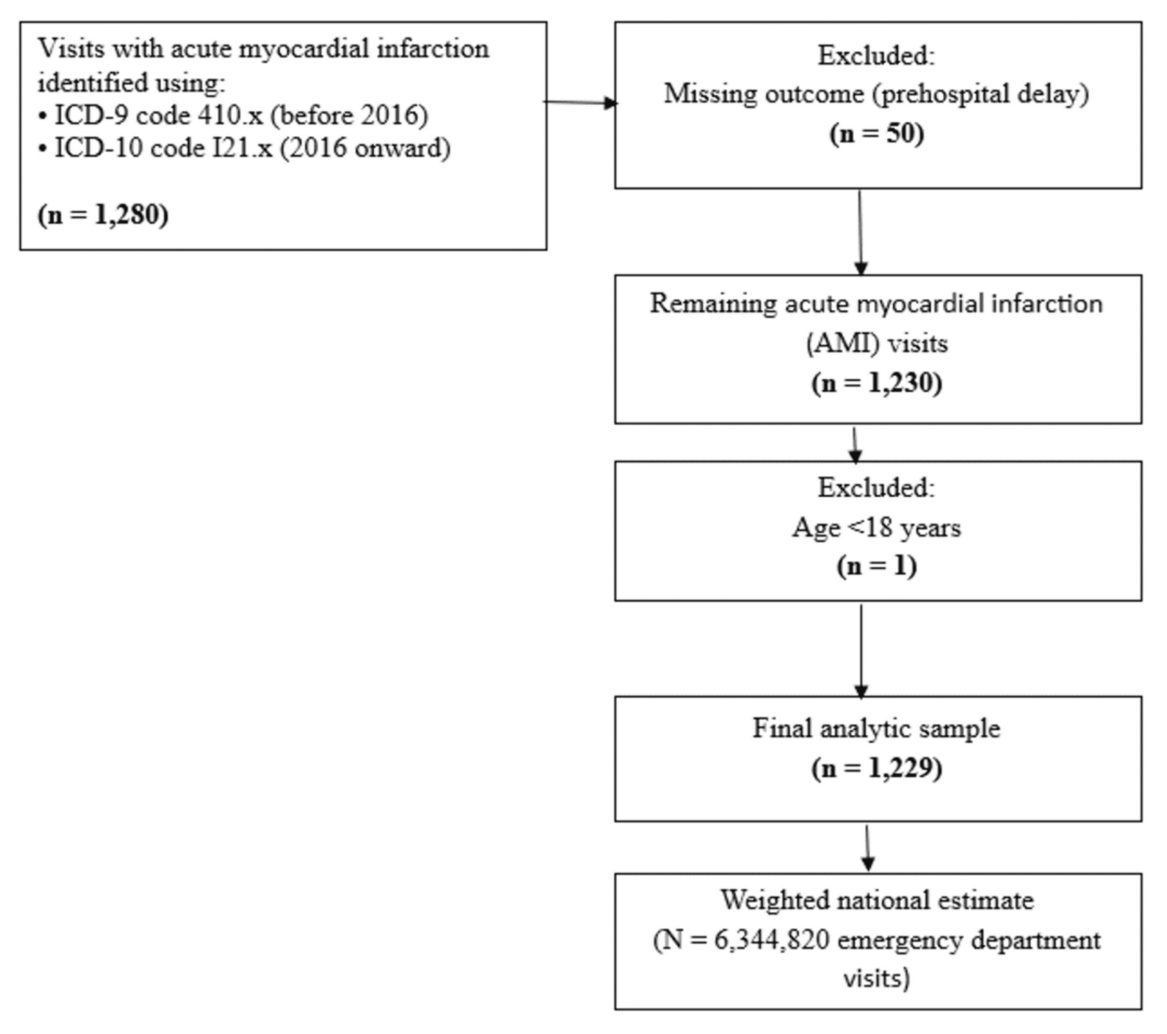
Flowchart of study population selection The notation ".x" indicates inclusion of all subcodes within the respective acute myocardial infarction diagnostic category. ICD-9: International Classification of Diseases, Ninth Revision; ICD-10: International Classification of Diseases, Tenth Revision

The study population included emergency department visits with a diagnosis of AMI. Cases were identified using the primary diagnosis field (DIAG1), secondary diagnosis field (DIAG2), and tertiary diagnosis field (DIAG3), based on International Classification of Diseases (ICD) codes recorded for each emergency department visit in the NHAMCS database [21]. AMI was identified using International Classification of Diseases (ICD )diagnostic codes recorded in the NHAMCS diagnosis fields. For survey years prior to 2016, cases were identified using ICD-Ninth Revision, Clinical Modification (ICD-9-CM) code 410 and all associated subcategories (410.x), whereas for 2016 onward, cases were identified using ICD-Tenth Revision, Clinical Modification (ICD-10-CM) code I21 and all associated subcategories (I21.x) [22]. The notation ".x" indicates inclusion of all diagnostic subcategories within the AMI code group. These codes have been widely used and validated for the identification of AMI in administrative health databases [22]. After identifying eligible visits, a total of 1,280 observations met the case definition. Observations with missing values in the outcome variable were excluded, resulting in the removal of 50 cases. The dataset was further restricted to adults aged 18 years and older, with one additional observation excluded. The final analytic sample included 1,229 unweighted observations, representing approximately 6,344,820 weighted emergency department visits, as shown in Figure 1 below. In NHAMCS, the unit of analysis is visits rather than individual persons, and the sampling weights are designed to generate nationally representative estimates of visit volume. The weighted population observed in this study reflects national estimates restricted to visits for AMI after applying inclusion criteria and survey design specifications, including stratification and clustering.

Variables and measures

The primary exposure variable was sex, categorized as male or female based on the NHAMCS sex variable. The primary outcome was a proxy measure of prehospital delay, approximated using triage urgency. Direct information on symptom onset to hospital arrival time was not available in NHAMCS. Therefore, triage urgency was used as a surrogate measure to differentiate visits presenting with greater versus lesser clinical urgency at emergency department arrival. Triage variables were harmonized across survey years using available fields, including IMMED (immediacy with which the patient should be seen), IMMEDR (immediacy with which the patient should be seen (unimputed)), and IMMEDF (immediacy with which the patient should be seen (formatted)). These variables represent the urgency of the patient’s condition at presentation and correspond to standardized five-level triage categories in NHAMCS. Across survey years, these fields capture whether the patient required immediate, emergent, urgent, semi-urgent, or non-urgent care. Prehospital delay was operationalized as a binary variable, where early presentation was defined as triage levels 1 or 2 and delayed presentation as triage levels 3, 4, or 5. Triage urgency reflects clinical severity at presentation and has been validated as a reliable measure of patient acuity in emergency department settings using standardized five-level triage systems [23].

Covariates included age categorized into three groups, less than 45 years, 45 to 64 years, and 65 years and older, race and ethnicity categorized into non-Hispanic White, non-Hispanic Black, Hispanic, and other groups, and insurance type categorized as Medicaid, private insurance, or other. Mode of arrival was initially extracted as a potential variable using available ambulance-related fields. However, due to substantial missingness of 71.14% and inconsistency across survey years, this variable was excluded from the final analytic models and is considered a limitation of the study.

Missing data

Missing data were evaluated for all variables included in the analysis. The outcome variable had 3.91% missing values, which were excluded through complete case analysis. Observations with missing weights were excluded from survey-weighted analyses but retained in unweighted analyses. Other variables included in the final models had no missingness after recoding and harmonization. Complete case analysis was applied for all regression models to ensure consistency in estimation.

Statistical analysis

Descriptive statistics were calculated to summarize the characteristics of the study population. Group comparisons by the outcome variable were conducted using survey-adjusted t-tests for continuous variables and survey-adjusted chi-square tests for categorical variables. The association between sex and prehospital delay was assessed using survey-weighted logistic regression models to account for the complex sampling design of NHAMCS. The multivariable model included sex as the primary independent variable and was adjusted for age category, race and ethnicity, and insurance type. Survey-weighted analyses were conducted using the patient visit weight variable provided by NHAMCS (Patient Visit Weight (PATWT)), which generates nationally representative estimates of emergency department visits. This weight was selected because it is consistently available across survey years and appropriate for visit-level analyses. Survey design was specified using strata and primary sampling unit variables provided in the dataset. Statistical significance was defined at a two-sided alpha level of 0.05. All analyses were conducted using Stata 18 (StataCorp LLC, College Station, TX, USA) [24].

Ethical considerations

This study used publicly available, deidentified data from NHAMCS. The dataset does not contain direct patient identifiers, and all information is anonymized prior to release. As a result, the study was exempt from institutional review board approval in accordance with standard guidelines for research using publicly available data.

## Results

Table 1 below presents the baseline characteristics of the study population stratified by sex.

**Table 1:** Baseline characteristics stratified by sex Values are survey-weighted counts and column percentages. The unweighted sample size was 1229 observations, representing 6,344,820 weighted emergency department visits. Group comparisons were performed using survey-adjusted chi-square tests for categorical variables. Percentages may not sum to 100% due to rounding. The asterisk (*) indicates statistical significance at p < 0.05. The symbol (–) indicates that the value is intentionally left blank. Race/ethnicity information is reported as documented in the source data. The table was generated by the authors using Stata version 18 (StataCorp LLC, College Station, TX, USA) [24].

Characteristic	Male (N = 2,570,936)	Female (N = 3,773,884)	Test statistic	p-value
Age Category, n (%)	–	–	F = 6.66	0.001*
<45 years	200,283 (7.8%)	453,544 (12.0%)	–	–
45–64 years	785,621 (30.6%)	1,597,760 (42.3%)	–	–
≥65 years	1,585,032 (61.7%)	1,722,580 (45.6%)	–	–
Race/Ethnicity, n (%)	–	–	F = 2.54	0.057
Non-Hispanic White	1,835,604 (71.4%)	2,787,168 (73.9%)	–	–
Non-Hispanic Black	392,597 (15.3%)	487,953 (12.9%)	–	–
Hispanic	297,631 (11.6%)	289,433 (7.7%)	–	–
Other	45,104 (1.8%)	209,329 (5.5%)	–	–
Insurance, n (%)	–	–	F = 0.87	0.416
Medicaid	445,467 (17.3%)	596,411 (15.8%)	–	–
Private	1,085,809 (42.2%)	1,775,987 (47.1%)	–	–

**Figure 2:**
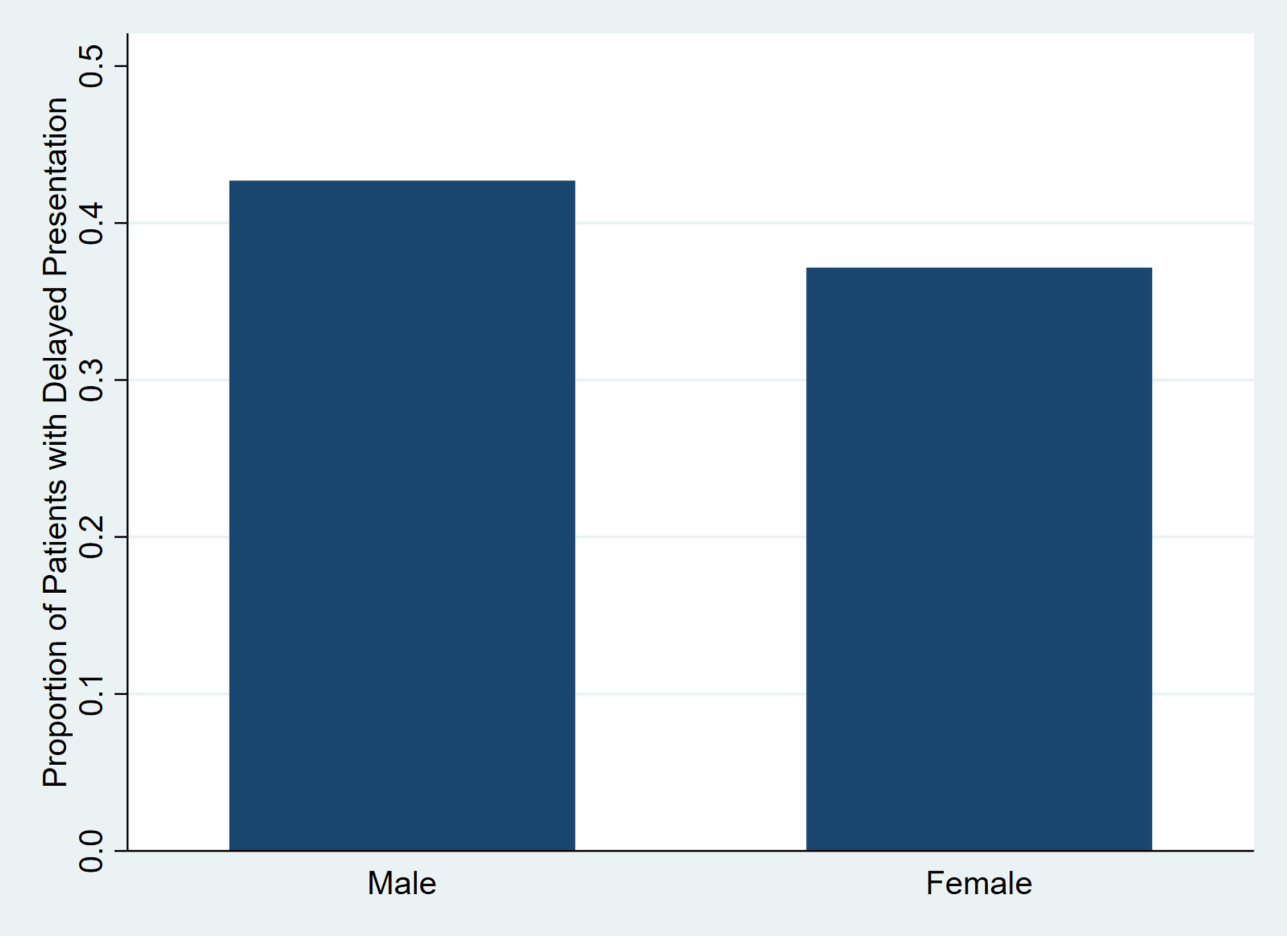
Proportion of patients with delayed presentation, stratified by sex Values represent survey-weighted proportions of patients classified as having delayed presentation. The y-axis shows the proportion of patients with delayed presentation. Estimates were derived using survey-weighted proportions accounting for clustering, stratification, and sampling weights. The figure was generated by the authors using Stata version 18 (StataCorp LLC, College Station, TX, USA) [24].

The results indicate differences in age distribution between males and females. Among males, 200,283 (7.8%) were younger than 45 years, 785,621 (30.6%) were aged 45 to 64 years, and 1,585,032 (61.7%) were aged 65 years and older. Among females, 453,544 (12.0%) were younger than 45 years, 1,597,760 (42.3%) were aged 45 to 64 years, and 1,722,580 (45.6%) were aged 65 years and older. The distribution of age categories differed significantly by sex (p = 0.001). For race and ethnicity, 1,835,604 (71.4%) of males and 2,787,168 (73.9%) of females were non-Hispanic White. Non-Hispanic Black individuals accounted for 392,597 (15.3%) of males and 487,953 (12.9%) of females. Hispanic individuals represented 297,631 (11.6%) of males and 289,433 (7.7%) of females, while other races accounted for 45,104 (1.8%) of males and 209,329 (5.5%) of females. These differences were not statistically significant (p = 0.057). For insurance type, Medicaid coverage was observed in 445,467 (17.3%) of males and 596,411 (15.8%) of females. Private insurance was more common among females at 1,775,987 (47.1%) compared to 1,085,809 (42.2%) among males. Other insurance types were reported in 1,039,660 (40.4%) of males and 1,401,485 (37.1%) of females. The distribution of insurance type did not differ significantly by sex (p = 0.416). Figure 2 illustrates the proportion of patients with delayed presentation by sex.

The results indicate that the proportion of delayed presentation was higher among males compared to females. Approximately 0.40 of males experienced delayed presentation, while the proportion among females was approximately 0.31. This pattern is consistent with the distribution observed in the descriptive analysis in Table 1. Table 2 presents the association between sex and prehospital delay.

**Table 2:** Association between sex and prehospital delay Results are from survey-weighted logistic regression models. Values are adjusted odds ratios with 95% confidence intervals. The model was adjusted for age category, race and ethnicity, and insurance type. The asterisk (*) indicates statistical significance at p < 0.05. The symbol (–) indicates that the value is intentionally left blank. Race/ethnicity information is reported as documented in the source data. The table was generated by the authors using Stata version 18 (StataCorp LLC, College Station, TX, USA) [24].

Variable	Adjusted odds ratio (95% CI)	p-value
Sex	–	–
Female vs Male	0.69 (0.51–0.93)	0.016*
Age Category	–	–
45–64 vs <45 years	1.03 (0.60–1.78)	0.907
≥65 vs <45 years	1.29 (0.72–2.30)	0.396
Race/Ethnicity	–	–
Non-Hispanic Black vs White	0.85 (0.54–1.36)	0.503
Hispanic vs White	1.19 (0.67–2.12)	0.547
Other vs White	0.88 (0.33–2.31)	0.790
Insurance	–	–
Private vs Medicaid	0.52 (0.32–0.85)	0.009*
Other vs Medicaid	0.42 (0.26–0.69)	<0.001*

The results indicate that female patients had lower odds of delayed presentation compared to male patients, with an adjusted odds ratio of 0.69 (95% CI 0.51 to 0.93, p = 0.016). Age category was not significantly associated with delayed presentation. Patients aged 45 to 64 years had an adjusted odds ratio of 1.03 (95% CI 0.60 to 1.78, p = 0.907), and those aged 65 years and older had an adjusted odds ratio of 1.29 (95% CI 0.72 to 2.30, p = 0.396), compared to those younger than 45 years. Race and ethnicity were also not significantly associated with delayed presentation. Non-Hispanic Black patients had an adjusted odds ratio of 0.85 (95% CI 0.54 to 1.36, p = 0.503), Hispanic patients had an adjusted odds ratio of 1.19 (95% CI 0.67 to 2.12, p = 0.547), and patients classified as other races had an adjusted odds ratio of 0.88 (95% CI 0.33 to 2.31, p = 0.790), compared to non-Hispanic White patients. Insurance type showed a significant association with delayed presentation. Patients with private insurance had lower odds of delayed presentation compared to those with Medicaid, with an adjusted odds ratio of 0.52 (95% CI 0.32 to 0.85, p = 0.009). Similarly, patients with other types of insurance had lower odds of delayed presentation, with an adjusted odds ratio of 0.42 (95% CI 0.26 to 0.69, p < 0.001).

## Discussion

In this study, the association between sex and prehospital delay among patients with AMI was examined using nationally representative emergency department visit data. The findings indicate that female patients had lower odds of the study-defined delayed presentation, based on triage acuity at emergency department arrival, compared to male patients after adjustment for age, race, ethnicity, and insurance type. A higher proportion of delayed presentation was observed among males in the descriptive analysis, and this difference remained statistically significant in the adjusted model. Age category and race and ethnicity were not significantly associated with delay, while insurance status showed a significant relationship, with privately insured and other insured patients demonstrating lower odds of delay compared to those with Medicaid. These findings are consistent with prior work showing differences in prehospital delay by sex, although the direction and magnitude of these differences have varied across settings [4,5]. Earlier studies have often reported longer delays among women, partly attributed to differences in symptom perception and healthcare-seeking behavior [6,7]. However, more recent analyses suggest that these patterns are not uniform and may reflect changes in awareness, access to care, and healthcare delivery over time [17]. The lower odds of delay observed among female patients in this study may reflect improvements in recognition of myocardial infarction symptoms among women or differences in healthcare utilization patterns.

Clinical guidelines emphasize the importance of early recognition and prompt treatment of AMI to improve outcomes. The American College of Cardiology and American Heart Association (ACCF/AHA) guideline recommends rapid identification and timely reperfusion therapy, highlighting that delays in presentation are associated with worse clinical outcomes [1]. Similarly, the European Society of Cardiology (ESC) guideline stresses minimizing total ischemic time through early patient presentation and efficient care pathways [2]. Evidence from clinical studies demonstrates that shorter time to treatment is associated with improved survival and reduced complications [3]. The findings from this study align with these recommendations by showing that delays in presentation remain common and differ across patient groups. The observed association between insurance type and delay may reflect differences in access to healthcare resources or health-seeking behavior, which can influence the timeliness of presentation.

Several factors may contribute to the observed differences in delay by sex. Differences in symptom recognition and interpretation have been widely reported, with women more likely to experience non-classic symptoms, which may affect the decision to seek care [9,10,11]. Variations in the perceived severity of symptoms, social roles, and prior healthcare experiences may also influence the timing of presentation [8,12]. Biological differences in disease presentation and progression have been suggested, although the extent to which these factors affect delay remains uncertain [14]. Healthcare system factors, including access to emergency services and prior interactions with healthcare providers, may further contribute to differences in presentation patterns [18]. The association between insurance status and delay observed in this study may reflect barriers related to cost, access, or continuity of care, which can influence the decision to seek timely medical attention.

Strengths and limitations of the study

This study has several strengths and limitations. The use of a large, nationally representative dataset allows for generalizable estimates of emergency department visits for AMI. The application of survey weights and adjustment for the complex sampling design strengthens the validity of the findings. However, the cross-sectional nature of the data limits the ability to assess temporal relationships. Most importantly, the study outcome was derived from triage acuity and does not directly measure the interval between symptom onset and hospital arrival. Consequently, outcome misclassification is possible, and the findings should be interpreted as associations with a proxy measure of prehospital delay rather than direct measurements of patient delay behavior. The study also relies on administrative and survey data, which may include measurement error and limited clinical detail. Residual confounding may remain due to unavailable variables such as symptom characteristics, infarction subtype, socioeconomic factors, and mode of transportation. Future research should focus on incorporating more detailed clinical variables and exploring longitudinal data to better understand factors influencing prehospital delay.

## Conclusions

This study highlights sex differences in a study-defined measure of delayed presentation among patients with AMI. Female patients showed lower odds of delayed presentation based on triage acuity compared to males after accounting for demographic and insurance factors. Because the outcome represents a proxy measure rather than a direct assessment of symptom onset to hospital arrival time, the findings should be interpreted cautiously. Further research using direct measures of prehospital delay is warranted.
